# Metagenomic Analysis of the Microbial Communities and Resistomes of Veal Calf Feces

**DOI:** 10.3389/fmicb.2020.609950

**Published:** 2021-02-09

**Authors:** Serajus Salaheen, Seon Woo Kim, Ernest Hovingh, Jo Ann S. Van Kessel, Bradd J. Haley

**Affiliations:** ^1^Environmental Microbial and Food Safety Laboratory, Beltsville Agricultural Research Center, Agricultural Research Service, United States Department of Agriculture, Beltsville, MD, United States; ^2^Department of Veterinary and Biomedical Sciences, Pennsylvania State University, University Park, PA, United States

**Keywords:** veal calves, resistome, metagenome, antimicrobial resistance, microbial community

## Abstract

Antimicrobial resistance (AMR) is a major public health concern, and dairy calves, including veal calves, are known reservoirs of resistant bacteria. To investigate AMR in the fecal microbial communities of veal calves, we conducted metagenomic sequencing of feces collected from individual animals on four commercial veal operations in Pennsylvania. Fecal samples from three randomly selected calves on each farm were collected soon after the calves were brought onto the farms (*n* = 12), and again, just before the calves from the same cohorts were ready for slaughter (*n* = 12). Results indicated that the most frequently identified phyla were Firmicutes, Bacteroidetes, Proteobacteria, and Actinobacteria. Fecal microbial communities in samples collected from the calves at the early and late stages of production were significantly different at the genus level (analysis of similarities [ANOSIM] on Bray-Curtis distances, *R* = 0.37, *p* < 0.05), but not at the phylum level. Variances among microbial communities in the feces of the younger calves were significantly higher than those from the feces of calves at the late stage of production (betadisper *F* = 8.25, *p* < 0.05). Additionally, our analyses identified a diverse set of mobile antimicrobial resistance genes (ARGs) in the veal calf feces. The fecal resistomes mostly consisted of ARGs that confer resistance to aminoglycosides, tetracyclines, and macrolide-lincosamide-streptogramin B (MLS), and these ARGs represented more than 70% of the fecal resistomes. Factors that are responsible for selection and persistence of resistant bacteria in the veal calf gut need to be identified to implement novel control points and interrupt detrimental AMR occurrence and shedding.

## Introduction

Currently there are approximately 9 million dairy cows in the United States, and on average, these animals calve every 13 months (U.S. Department of Agriculture [USDA], 2016a; U.S. Department of Agriculture [USDA], 2018). Female calves are usually raised as replacement heifers, but male calves are typically sold as bob veal or for dairy beef or veal production. The latter are typically milk-fed and are raised to approximately 16–18 weeks of age or around 450 lbs., and the meat is marketed as veal. Veal calves lack functional rumens and are raised under a different nutritional management program than calves raised as replacements for the milking herd. In 2015, close to a half million veal calves were slaughtered under federal inspection in the United States (U.S. Department of Agriculture [USDA], and Agricultural Marketing Service [AMS], 2015). Production wastes from livestock, including veal calves, harbor both pathogenic and resistant bacteria, but studies on the microbial communities and their resistomes in veal calf waste are lacking (Um et al., 2016; Salaheen et al., 2019).

Veal calves often face health and disease challenges from transportation stress, comingling, adaptation to new housing arrangements, dietary changes, and exposure to pathogens from multiple sources. Antimicrobials may therefore be administered to groups of veal calves as a preventive measure or to treat disease outbreaks. In 2014, before the full implementation of the Veterinary Feed Directive, 37.6% of the United States dairy operations fed at least some of their preweaned calves medicated milk replacers (U.S. Department of Agriculture [USDA], 2016a). In the United States, antimicrobials are administered to preweaned dairy calves mainly to treat digestive diseases (mostly tetracyclines, third generation cephalosporins, and trimethoprim/sulfamethoxazole), respiratory diseases (mostly macrolides and florfenicol), and navel infections (primarily penicillin) (U.S. Department of Agriculture [USDA], 2016b). Although residue violation rates for dairy cows and bob veal (calves sold from the herd and slaughtered directly; generally less than a week old) has recently decreased, these two groups accounted for 85% of the violations reported under the inspector-generated sampling plan (U.S. Department of Agriculture [USDA], and Food Safety and Inspection Service [FSIS], 2019).

Antimicrobial resistance (AMR) has been attributed to the use of therapeutic and subtherapeutic antimicrobials in food animal production (Langford et al., 2003; Ghosh and LaPara, 2007; Kennedy, 2013). However, resistant bacteria have also been identified in untreated animals and environments that were not anthropogenically impacted (Khachatryan et al., 2004; Allen et al., 2010; Bhullar et al., 2012; Cytryn, 2013; Liu et al., 2019a). Currently veal calves are mostly raised in group pens potentially allowing transfer of resistant bacteria between calves and maintenance within the group. Repeated influx of calves from multiple sources and other environmental sources may promote long-term persistence of resistant bacteria in the farm environment and may increase the probability of transmission to and colonization of naïve non-comingled animals (Liu et al., 2016, 2019b). In addition to the potential risk for human consumption of contaminated product and exposure of farm personnel and their families to resistant bacteria, farm effluent can potentially transmit these resistant bacteria into aquatic, atmospheric, and terrestrial ecosystems (McEachran et al., 2015; Sura et al., 2015; Um et al., 2016).

The prevalence of resistant bacteria in dairy animals is age-dependent with a higher abundance in calves, especially pre-weaning, than adult cows and seems to be unrelated to recent antimicrobial use (Hoyle et al., 2004; Khachatryan et al., 2004; Cao et al., 2019; Springer et al., 2019; Haley et al., 2020). In a culture-dependent study, we previously observed that multidrug resistant (MDR, resistant to more than three antimicrobial classes) generic *Escherichia coli* were more prevalent in fecal samples from veal calves at slaughter-age compared with the calves from the same cohort approximately 2 months earlier (Salaheen et al., 2019). Dill-McFarland et al. (2017) reported that development of the gut microbiota in dairy animals is age-dependent from birth to adulthood, and the dynamic changes in the resistome structure may be closely related to the gut microbiota in dairy calves (Liu et al., 2019b).

Currently, there is very limited information available on carriage and distribution of AMR in the United States veal industry. Therefore, the goal of this study was to characterize the fecal microbial community and resistome structures of commercially raised veal calves in early and late stages of production.

## Materials and Methods

### Sample Collection, Processing, and Analysis

Fecal samples were previously collected from individual calves on commercial veal farms in Pennsylvania (Salaheen et al., 2019). Veal calf management protocols were not made available. Sampling procedures were approved by the Institutional Animal Care and Use Committee (IACUC, protocol number 42381-1). Each farm was visited twice, soon after the calves were brought onto the farms (typically less than 2 weeks of age) and again shortly before the animals were sent to slaughter (2–3 months). The second samplings were collected from the same cohorts, but not necessarily the same calves, as the first samplings. Fecal samples were shipped on ice, suspended in buffered peptone water (10 g in 20 mL) in a filtered bag. They were mixed with a BagMixer (Interscience, Woburn, MA), and two milliliters of filtrate were stored at −80°C. A total of 24 filtrates from four farms (three filtrates from each farm during the first samplings, and again three filtrates from the same farms during the second samplings: in total 12 filtrate pairs from first and second samplings) were randomly selected for metagenomic sequencing. Filtrates were thawed on ice and DNA was extracted with MoBio PowerSoil^®^ DNA Isolation Kit (Carlsbad, CA, United States) and cleaned by using a DNA Clean and Concentrator kit (Zymo Research, Irvine, CA, United States). DNA was sheared by using a Covaris-focused ultrasonicator (Covaris, Woburn, MA, United States). DNA libraries were prepared for each of the 24 samples using a TruSeq Nano Kit (Illumina, San Diego, CA, United States) on the NeoPrep Library Prep system. Paired-end sequencing (2 × 150 bp reads) was conducted with a high-output flow cell on a NextSeq500 sequencing platform (Illumina). Metagenomic sequence data have been deposited at NCBI under accession numbers SRX8728194, SRX8728195, SRX8728206, SRX8728211, SRX8728212, SRX8728213, SRX8728214, SRX8728215, SRX8 728216, SRX8728217, SRX8728196, SRX8728197, SRX8728198, SRX8728199, SRX8728200, SRX8728201, SRX8728202, SRX8 728203, SRX8728204, SRX8728205, SRX8728207, SRX8728208, SRX8728209, and SRX8728210.

### Analysis of Metagenomic Datasets

Sequence data were demultiplexed using the BCL2FastQ V 2.15.0.4 (Illumina). PhiX, sequencing adaptors, and host (UMD 3.1 *Bos Taurus* genome) specific reads were removed using DeconSeq V 0.4.3 (Schmieder and Edwards, 2011). Reads were further trimmed using Trimmomatic V 0.36 (leading 20, trailing 20, sliding 4:20, and min len 36) (Bolger et al., 2014). To investigate the taxonomic profiles, 16S rRNA sequences were identified using Metaxa2 (Bengtsson-Palme et al., 2015) with default parameters and taxonomies were then assigned using EzBioCloud server (Yoon et al., 2017). Taxonomic diversity information was collected from the EzBioCloud server output and taxonomic abundances were normalized by dividing the number of reads of a specific taxon by the total number of reads assigned to 16S rRNA in that sample.

Interrogation of sequence reads for identity to acquired antimicrobial resistance genes (ARGs) was performed by aligning the reads from each metagenome to the ResFinder database (downloaded on 05/01/2019; Zankari et al., 2012) using BLASTn V 2.7.1 (sequence identity ≥95%, matched sequence length ≥50 bp). Percent sequence identity and gene length coverage were used to determine the best-hit on the BLASTn analysis. A minimum of 10 reads assigned to an ARG was the cutoff to consider presence in a sample (Haley et al., 2020). Relative abundances of ARGs were normalized using reference gene length and expressed as “ARGs per copy of 16S rRNA gene” as conducted by Li et al. (2015) using the formula:

(1) $$\text{Abundance}=\sum_{1}^{n}\frac{\text{N}\,\left(\mathrm{ARG}\,\mathrm{sequence}\right)\times \text{L}\,\left(\text{reads}\right)/L\,\left(\mathrm{ARG}\,\mathrm{reference}\,\mathrm{sequence}\right)}{\text{N}\,\left(16\,S\,\mathrm{rRNA}\,\mathrm{sequence}\right)\times \text{L}\,\left(\text{reads}\right)/L\,\left(16\,S\,\mathrm{rRNA}\,\mathrm{sequence}\right)}$$

where n stands for the total number of ARGs; N(ARG sequence) is the number of ARG-specific reads mapped to the ResFinder database; L(reads) is the length of the sequencing reads; L(ARG reference sequence) is the length of the corresponding reference sequence in the ResFinder database; N(16S rRNA sequence) is the number of reads mapped to 16S rRNA genes determined by Metaxa2; L(16s rRNA sequence) is the average length (1,427 bp) of a 16S rRNA gene sequence in the EzBioCloud database.

Differential abundances in taxa and ARGs between groups (samples from calves at younger and older ages) were determined by linear discriminant analysis (LDA) coupled with effect size measurements (LEfSe) [alpha values for the Kruskal-Wallis and pairwise Wilcoxon tests were set to 0.05, pairwise Wilcoxon tests were conducted between samples within farms, and threshold on the logarithmic LDA score for discriminative features was set to 2.0] (Segata et al., 2011). Differential abundances in taxa and ARGs between groups were further evaluated by supervised machine learning with the randomForest package in R (Liaw and Wiener, 2002).

To compare compositions of the resistomes and bacterial communities, non-metric multi-dimensional scaling (NMDS) on Bray-Curtis distance between each sample (based on relative abundances of taxa or ARGs) were performed with the vegan package in R (Oksanen et al., 2019). Analysis of similarities (ANOSIM), and homogeneity of multivariate dispersions were carried out with the “anosim” and “betadisper” functions in vegan, respectively. Wilcoxon tests on the alpha-diversity indices and relative abundances of ARGs were conducted using calf as the experimental unit and farm as a blocking factor on JMP Pro 13 (SAS Institute, Inc., Cary, NC, United States). When appropriate, *p*-values were adjusted for multiple comparisons using the Benjamini-Hochberg (BH) method. BH-adjusted *p*-values (P*_*adj*_*) less than 0.05 were considered statistically significant.

To assess the potential for co-occurrence of ARGs that were present in at least 50% (12 of 24) of the metagenomes, a correlation matrix was generated by calculating all possible pairwise Spearman’s rank correlations between the ARGs. This preliminary filtering step eliminated the ARGs that were represented in a limited number of samples and thus reduced artificial correlation biases. Correlations between the ARGs with a Spearman’s correlation coefficient (ρ) value of ≥0.80 and a *p*-value of <0.01 was considered valid, hence included in the analysis (Li et al., 2015). The co-occurrence patterns among ARGs were then explored on an interactive network inference, Gephi version 0.9.1 (Bastian et al., 2009).

## Results

### General Information on Taxonomic Affiliation

To assess the microbial composition of veal calf feces, a total of 24 fecal samples from individual calves were analyzed using shotgun metagenomic sequencing. The total number of cleaned and curated reads in each metagenome ranged between 24.5 and 127.7 M (median: 63.8 M). The percentages of reads that were assigned to the host genome (*Bos Taurus*) ranged between 0.2 and 22.5% (median: 0.3%). In total, 1,044,241 bacterial 16S rRNA sequences were detected from the 24 metagenomes (range: 13,483 to 67,381, median: 45,753). An increased proportion of unclassified sequences were observed with descending taxonomic hierarchy. In total, 35, 351, and 1,370 classified/known phyla, families, and genera, respectively, were detected. Members of four phyla, Actinobacteria, Bacteroidetes, Firmicutes, and Proteobacteria, were identified among all 24 metagenomes. In total, 5,115 bacterial Operational Taxonomic Units (OTUs, 97% sequence similarity threshold) were detected. Evaluation of the individual rarefaction curves on rarefied numbers of bacterial OTUs indicated that all 24 curves tended to plateau (data not shown).

### The Fecal Microbial Communities

To compare the fecal microbial communities of veal calves at different ages, the 24 metagenomes were divided into two groups: (i) fecal samples that were collected from calves at early stage of production soon after they were brought onto the farms (Sampling 1) and (ii) fecal samples from the same cohort of calves at slaughter-age/late stage of production (Sampling 2).

**FIGURE 1:**
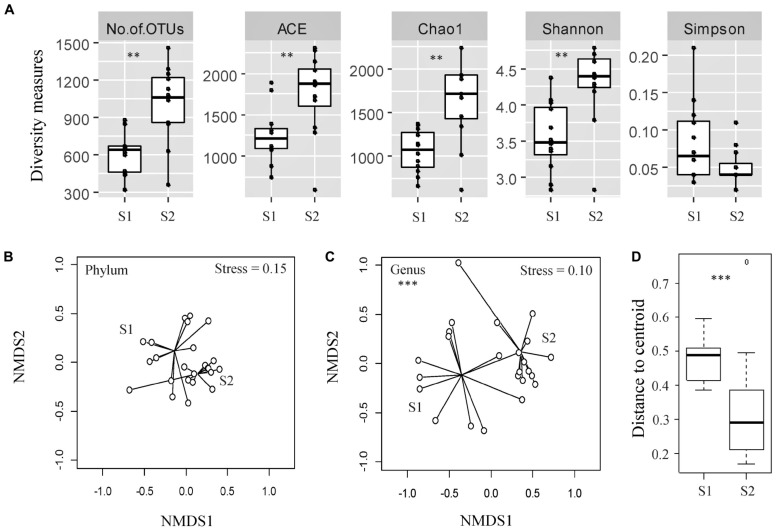
Assessment of alpha- and beta-diversity of fecal samples taken from the same cohort of veal calves at early (Sampling 1/S1, *n* = 12) and late (Sampling 2/S2, *n* = 12) stages of production. **(A)** Alpha-diversity measures of individual samples from calves from same cohort at S1, S2. **(B,C)** Non-metric multidimensional scaling (NMDS) plots based on Bray-Curtis distance matrix encompassing 24 datasets from the two stages of veal production. **(D)** Distance to centroid from the multivariate homogeneity of groups dispersions test. Triple asterisks (^∗∗∗^) represent statistical significance with *p* < 0.001, double asterisks (**) represent *p* < 0.01, a single asterisk (*) represents *p* < 0.05, and no asterisk represents no observed statistical significance.

The diversity of bacterial OTUs in individual fecal samples was measured using ACE (estimator of species richness), Chao1 (estimator of species richness), Shannon (estimator of both species richness and evenness with more weight on low abundant taxa), and Simpson (estimator of both species richness and evenness with more weight on dominant taxa) indices (Figure 1A). Fecal samples from the calves at an older age exhibited significantly higher OTU richness compared with the samples from the same cohort taken at a younger age, as measured by the ACE and Chao1 indices (*p* < 0.05). Samples from the calves at an older age also displayed significantly higher diversity, likely due to rare/low abundant taxa (Shannon index, *p* < 0.05) but not due to dominant taxa (Simpson index, *p* > 0.05).

The NMDS ordination plot and an ANOSIM indicated that the overall fecal microbial communities were significantly different between samples collected from the calves during two stages of production at the genus level, but not at the phylum level (ANOSIM R = 0.37 and 0.04, *p* < 0.05 and > 0.05, Stress = 0.10 and 0.15 at the genus and phylum levels, respectively; Figures 1B,C). There were significant differences between microbial community structures of veal calf feces from different farms at both stages of production (Supplementary File 1). Based on the NMDS analysis, samples from the calves at an older age appeared to cluster more closely to each other than the samples from calves at a younger age, in ordination space. Multivariate homogeneity of groups dispersions test indicated that the dispersions (variances) between different samples from calves at a younger age were significantly higher than the samples from the same cohort of calves at an older age (betadisper *F* = 8.25, *p* < 0.05; Figure 1D).

**FIGURE 2:**
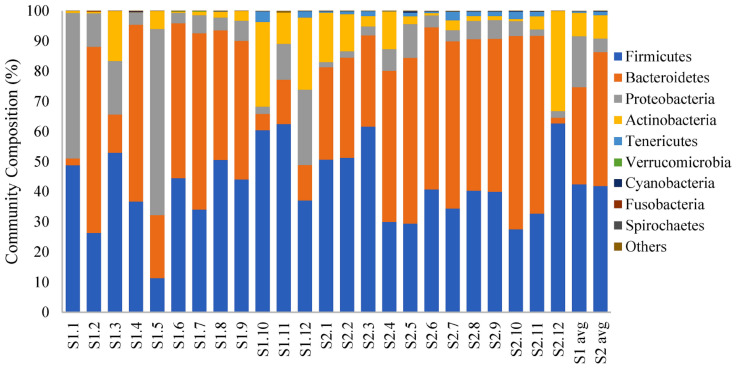
100% stacked bar plot showing relative abundance (%) of bacterial phyla in fecal samples collected from veal calves soon after they were brought onto the farms (Sampling 1/S1, *n* = 12) and fecal samples from the same cohort of calves at slaughter-age (Sampling 2/S2, *n* = 12). The last two columns are the average community composition for samples from the two stages of veal production.

Firmicutes, Bacteroidetes, Proteobacteria, and Actinobacteria were the most frequently identified phyla in both groups (Figure 2). Relative abundances of the two major phyla, Firmicutes and Bacteroidetes, remained more comparable across the fecal microbial communities of the calves at an older age compared with the same cohort of calves at a younger age. In total, Firmicutes and Bacteroidetes constituted at least 80% of the bacterial communities in the feces of 11 (of 12) calves sampled close to slaughter compared with 6 (of 12) calves at a younger age. Members of the phylum, Proteobacteria, constituted more than 10% of the identified phyla in half of the samples taken from the younger calves but in only one fecal sample from an older calf (Sampling 1: 2.40 to 61.79%, median = 8.89%; Sampling 2: 1.71 to 11.20%, median = 3.84%). However, using LDA coupled with effect size measurements, we did not identify any phyla that were differentially abundant in the fecal samples from the calves at the two stages of production. At the genus level, there were nine classified genera that were more abundant in younger calf feces and 47 genera that were more abundant in the feces collected from calves ∼ 2 months later (Supplementary File 2).

### The Fecal Resistomes

**FIGURE 3:**
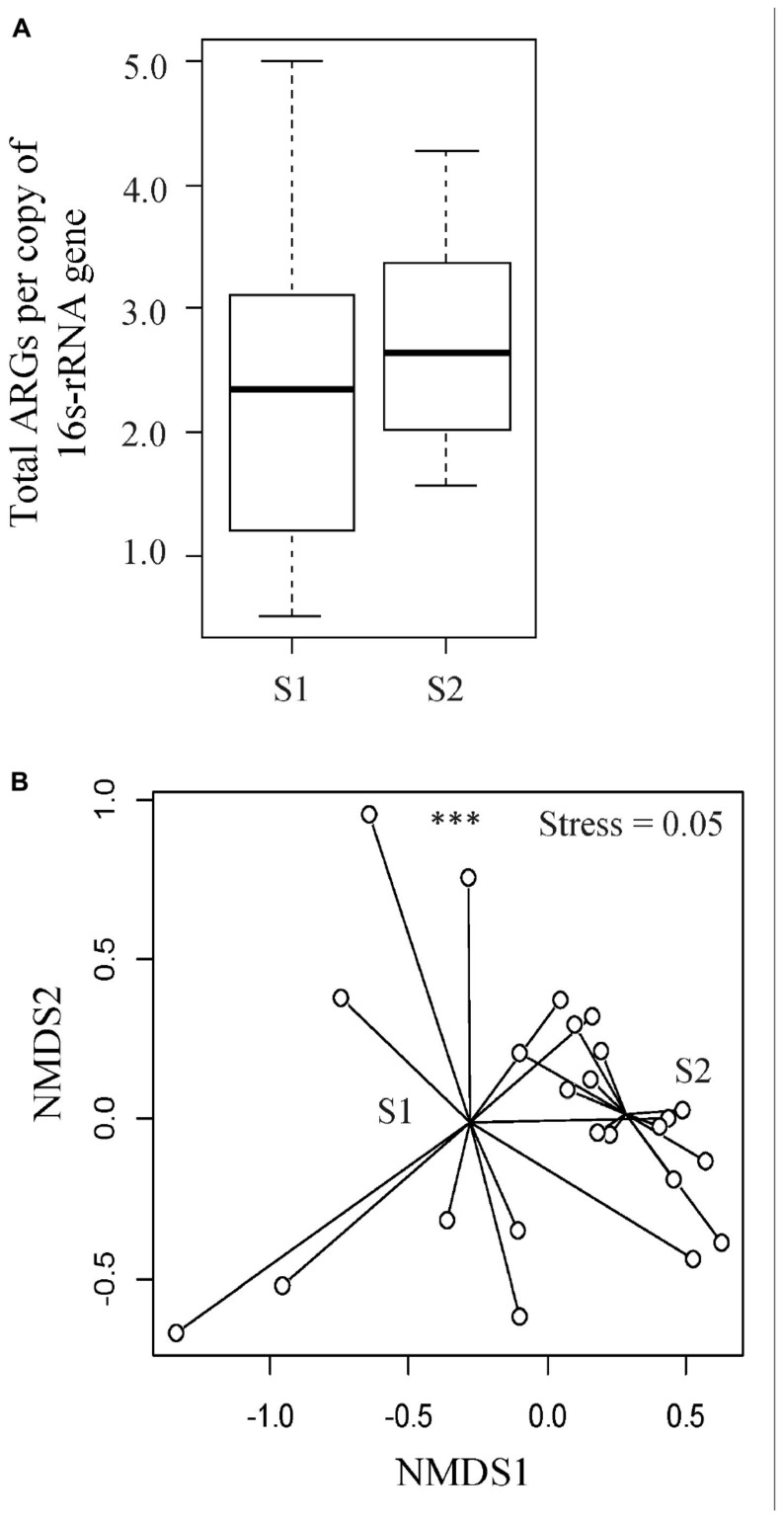
Evaluation of resistome profiles in feces collected from veal calves soon after they were brought onto the farms (Sampling 1/S1, *n* = 12) and fecal samples from the same cohort of calves at slaughter-age (Sampling 2/S2, *n* = 12). **(A)** Boxplots of the relative abundances of 16S rRNA-normalized ARGs. **(B)** Non-metric multidimensional scaling (NMDS) plot based on Bray-Curtis distance matrix of ARG families from the two stages of veal production. Triple asterisks (^∗∗∗^) represent statistical significance with *p* < 0.001, double asterisks (^∗∗^) represent *p* < 0.01, a single asterisk (^∗^) represents *p* < 0.05, and no asterisk represents no statistical significance were observed.

To understand the diversity and dynamics of the ARGs harbored by the veal calves’ fecal microbiota, metagenomic sequences were aligned to the ResFinder database and acquired ARGs were identified. In total, 150 ARG families (e.g., *bla*_*ACT*_, *bla*_*TEM*_) and 12 ARG classes (e.g., β-lactam, MLS, tetracycline resistance genes) were detected. The relative abundance of ARGs ranged between 0.51 and 5.00 ARG/16s rRNA (median = 2.3 ARG/16S rRNA) in the feces from young calves, and 1.56 to 4.28 ARG/16s rRNA (median = 2.6 ARG/16S rRNA) at an older age (Wilcoxon *p* > 0.05; Figure 3A). Among the ARG families, 12 were unique to the fecal samples from young calves, and 37 were unique to feces from the calves sampled near slaughter, with 101 identified in samples from both time points. We did not identify significant differences in ARG abundances in feces from calves when they were sampled soon after arrival on the farm or when they were close to slaughter, but the resistome composition differed significantly between the two time points (ARG family: ANOSIM *R* = 0.24, *p* < 0.05, Stress = 0.05; Figure 3B). Similar to the taxonomic profiles, dispersions (variances) between fecal resistomes of the calves at a younger age were significantly higher than that of the calves from the same cohorts when they were older (betadisper *F* = 23.04, *p* < 0.05).

**FIGURE 4:**
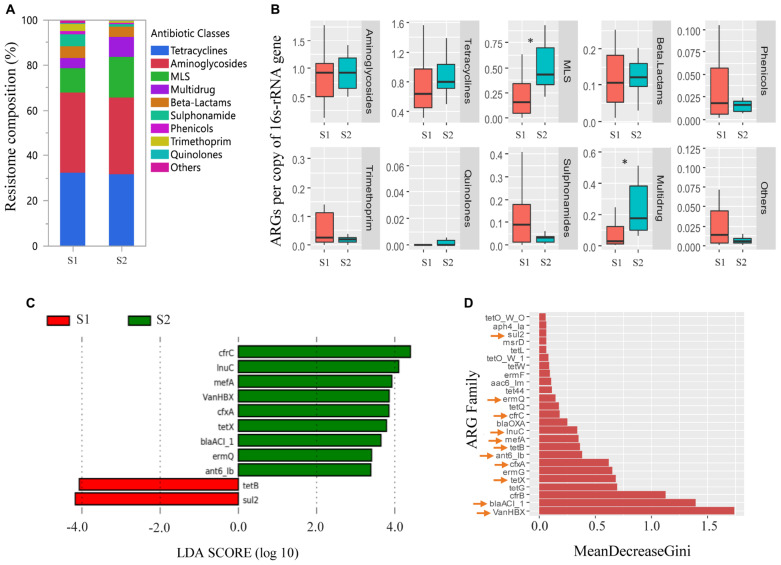
Antimicrobial resistance genes (ARGs) in feces collected from veal calves soon after they were brought onto the farms (Sampling 1/S1, *n* = 12) and fecal samples from the same cohort of calves at slaughter-age (Sampling 2/S2, *n* = 12). **(A,B)** Compare relative abundances of ARG classes in feces taken from calves at S1, S2. Antimicrobial classes: Fosfomycins, Polymyxins, and Glycopeptides constituted the “Others” group. **(C)** Differentially abundant ARGs in feces taken from calves at S1, S2 using linear discriminant analysis coupled with effect size measurements (LEfSe). **(D)** Differentially abundant ARGs in feces taken from calves at S1, S2 using randomForest. ARGs with an arrow are the ARGs that were found to be differentially abundant between the two samplings using LEfSe analysis. Triple asterisks (^∗∗∗^) represent statistical significance with *p* < 0.001, double asterisks (^∗∗^) represent *p* < 0.01, a single asterisk (^∗^) represents *p* < 0.05, and no asterisk represents no statistical significance were observed.

Overall, the fecal resistomes of veal calves were dominated by ARGs that conferred resistance to aminoglycosides, tetracycline, and MLS antimicrobials, which made up more than 70% of the resistomes (Figure 4A). There was a higher relative abundance of MLS and multidrug resistance genes (*cfrB, cfrC*, and *optrA*) in the feces of the calves at an older age (*p* < 0.05; Figure 4B). LEfSe analysis indicated that there were 11 differentially abundant ARG families, where nine were more abundant in the samples collected from the calves at an older age and two were more abundant in the samples taken from the calves at a younger age (Figure 4C). To increase confidence in the result and minimize potential biases, differential abundances in ARG families were further evaluated by a supervised machine learning approach with randomForest (Figure 4D). All of the 11 differentially abundant ARG families (from the LEfSe analysis) were also classified as important group-separators (calves at younger versus older age-time points) by the randomForest analysis. Genes that were more abundant in feces collected when the calves were close to slaughter included multiple drug classes consisting mostly of MLS and β-lactam resistance genes.

### Co-occurrence of ARG Families

**FIGURE 5:**
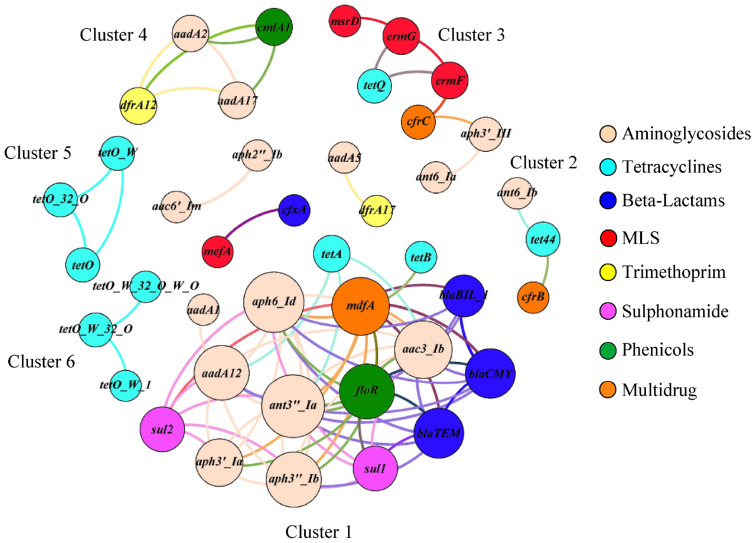
The co-occurrence network among ARG families in veal calf feces. The nodes (ARG families) were colored by the corresponding class of antimicrobials and the size of each node represents the number of connections or edges (degree). An edge represents a strong (Spearman *ρ* ≥ 0.8) and significant (*p* < 0.01) correlation between two ARG families and edges were colored according to their connecting nodes for better separation.

The correlation network consisted of 42 nodes (ARG families) and 84 edges (an edge represents a valid correlation between two ARG families) and formed a modular structure (modularity score 0.46; Figure 5) (Barberán et al., 2012). Modularity is a characteristic of network structures that quantifies the extent to which nodes group into modules (also termed as clusters or subnetworks). A module of highly interconnected nodes may represent a group of ARGs that originated from either a single microorganism or a group of microorganisms that share the same ecological niche with/without direct interaction (Steele et al., 2011; Barberán et al., 2012; Li et al., 2015). In this study, six clusters (with at-least three nodes) were identified that contained ARG families that conferred resistance to multiple antimicrobial classes. For example, ARG families conferring resistance to aminoglycosides (*ant6-Ia* and *aph3*′*-III*), tetracyclines (*tetQ*), MLS (*ermF*, *ermG*, and *msrD*), and multidrugs (*cfrC*) constituted Cluster three of the correlation network.

## Discussion

In a longitudinal study of twelve commercial veal operations, we isolated phenotypically diverse resistant and MDR generic *E. coli* from veal calf feces (Salaheen et al., 2019) including *E. coli* resistant to all of the 14 antimicrobials on the NARMS GN panel (CMV3AGNF, ampicillin, amoxicillin-clavulanic acid, cefoxitin, ceftiofur, ceftriaxone, gentamicin, streptomycin, sulfisoxazole, trimethoprim-sulfamethoxazole, tetracycline, chloramphenicol, azithromycin, ciprofloxacin, and nalidixic acid). Resistant *E. coli*, including MDR *E. coli*, were more prevalent in fecal samples taken from veal calves at slaughter-age compared with the calves from the same cohorts when they were just brought onto the farms. As a follow-up, we sequenced the metagenomes of a subset of the individual fecal samples to characterize the ARGs in the fecal microbial communities in these veal calves. We specifically focused on the acquired ARGs as these genes can potentially be transferred to pathogenic bacteria and are hence a possible public health concern. Our analyses identified diverse acquired/transferrable ARGs, including genes that confer resistance to the World Health Organization (WHO) classified “critically important antimicrobials” for humans (The World Health Organization [WHO], 2018). Fecal resistomes in the veal calf feces mostly consisted of ARGs that confer resistance to aminoglycosides, tetracyclines, and MLS; these ARGs represented more than 70% of the resistomes. Similarly, these three classes of ARGs were also found to dominate the fecal resistomes of preweaned dairy calves (Haley et al., 2020). Co-occurrence network analysis revealed that ARGs from multiple antimicrobial classes co-occurred in the veal calf fecal resistomes. This observations suggests possible MDR occurrence which is supported by our previous study where generic *E. coli* strains were isolated with AMR patterns like AMP-AZI-CHL-STR-FIS-TET-SXT (Ampicillin-Azithromycin-Chloramphenicol-Streptomycin-Sulfisoxazole-Tetracycline-Trimethoprim-Sulfamethoxazole) from veal calf feces (Salaheen et al., 2019). Occurrence of ARGs that potentially confer resistance to medically important antimicrobials, for example, colistin (*mcr*), extended spectrum β-lactams (*bla*_*CTX*_, *bla*_*OXA*_, *cepA*), fosfomycin (*fosA*), trimethoprim (*dfrA*), vancomycin (*vanHBX*), and multiple drug classes (*optrA, cfrB*, and *cfrC*) is of major concern.

Factors influencing the high prevalence of AMR in veal calves have not been fully described. Colostrum is a potential source of early-life ARGs in dairy calf resistomes that go through dynamic changes with transition in diet (Liu et al., 2019a, 2020). The farm environment also serves as a potential source of resistant bacteria that can become resident in the calf gut, but for this complex source it is difficult to identify the drivers of increased resistance in the calf gut. Antimicrobials are critical to prevent, control, and treat diseases in farm animals, but usage is regulated by the Food and Drug Administration (FDA). FDA-approved antimicrobials are used in veal production under veterinary oversight following the labeled dosage, route of administration, and withdrawal time (American Veal Association, 2019; National Milk Producers Federation, 2019). Antimicrobial usage may select for resistant bacteria in the calf gut; however, several studies have concluded that prevalence of resistant bacteria or ARGs may not necessarily be solely attributed to recent use of antimicrobials (Khachatryan et al., 2004; Thomas et al., 2017; Vikram et al., 2017; Liu et al., 2019a). It is highly likely that the mechanism of AMR carriage in veal calves is multifactorial. Nutritional management of calves raised for veal and calves raised as herd replacements is very different. Replacement calves are transitioned completely to solid feed by 6 to 8 weeks of age. Therefore, the gut ecology is significantly different between these two animal groups and that may play a role in the persistence of resistant bacteria in the veal calf gut. Several potential factors, such as co-selection for bacterial genomic traits linked to AMR phenotypes, competition between resistant and susceptible bacteria in the calf gut, and calf physiology and immunity, should be investigated.

We observed significant differences in the microbial communities of feces collected from veal calves that were recently brought onto the farms compared with feces taken from the same cohort approximately 2 months later at slaughter age. Although the relative abundances of the dominant taxa remained similar between the two sampling points, significant differences in the rare/low abundant taxa were observed. Possible factors influencing the age-related changes in the intestinal microbiota of the veal calves include, but may not be limited to, age (alteration of the gut physiology, maturation of immune system, stress, and other unidentified factors), diet (e.g., prior colostrum consumption, milk or milk replacer, grain and forages, water, and electrolytes), housing (group pens), microbial ecology of calves entering the herd, and the environment, and other management factors such as antimicrobial usage. Additionally, our observations of higher intragroup variability and less diversity in the fecal microbial communities of calves at a younger age compared to the calves at slaughter age concur with previous reports (Jami et al., 2013; Klein-Jöbstl et al., 2014; Dill-McFarland et al., 2017; Haley et al., 2020). Increased microbial diversity and intra-group similarity with age indicate that the fecal microbial communities converged toward a mature state in the veal calf cohorts over time.

Previously we observed that resistant and MDR *E. coli* were more prevalent in feces collected from calves just prior to slaughter compared with feces collected from younger calves as well as composite fecal samples from bob calves (Salaheen et al., 2019). In this study, the fecal resistome compositions of calves from the same cohort at younger and older ages differed significantly, and a number of ARGs were more abundant in the older calves. Maynou et al. (2017) reported that the prevalence of resistant *E. coli* was influenced by age in dairy calves, regardless of feeding regimens and Liu et al. (2019a) observed an overall increase in the diversity of transferrable ARGs in dairy calves over time, even in the absence of antimicrobial treatment. The increased diversity of transferable ARGs may be due to an accumulation of new species in the intestinal tracts of the calves with age. Veal calves are raised in group pens, so calf to calf transfer of resistant bacteria within pens may also increase the prevalence of resistant bacteria in the herd over time. However, the link between AMR occurrence and age is not completely understood. Usage of antimicrobials may also select for resistant bacteria, but we were unable to acquire data on the administration of antimicrobials in the herds that were sampled for this study.

Results from this study confirm previous culture-based analysis and demonstrate that commercially raised veal calves harbor and shed significant levels of bacteria that are resistant to a range of antimicrobials. In addition, there is a diversification of the fecal microbial communities and resistome structures in veal calves as they get older. Resistant and pathogenic bacteria that are shed in the feces pose a significant public health risk due to potential contamination of the final product, despite significant efforts to mitigate contamination in the slaughter process. Additional risks may occur due to environmental contamination or to direct exposure of the farm workers. Future work should focus on identifying management practices that may impact selection or enrichment of AMR. Factors that are responsible for selection and persistence of resistant bacteria in the veal calf gut need to be identified to discover novel control points to mitigate detrimental AMR occurrence and shedding.

## Data Availability Statement

The datasets presented in this study can be found in online repositories. The names of the repository/repositories and accession number(s) can be found in the article/Supplementary Material.

## Ethics Statement

The animal study was reviewed and approved by the Institutional Animal Care and Use Committee protocol number 42381-1.

## Author Contributions

JV, BH, and EH contributed to the conception and design of the work, ensuring that questions related to the accuracy or integrity of any part of the work are appropriately investigated and resolved, critically revised the final version to be published, and were responsible for the integrity of the work. SK contributed by performing metagenomic sequencing. SS contributed by analyzing and interpreting the data, and drafting and revising the manuscript. EH enrolled the farms and collected the fecal samples. All authors contributed to the article and approved the submitted version.

## Conflict of Interest

The authors declare that the research was conducted in the absence of any commercial or financial relationships that could be construed as a potential conflict of interest.
