# Supplementary material for: Metagenomic Analysis of the Microbial Communities and Resistomes of Veal Calf Feces
Source: Front Microbiol. 2021 Feb 9;11:609950. doi: 10.3389/fmicb.2020.609950 (PMC7899987; doi:10.3389/fmicb.2020.609950)
Supplement: Supplementary file 1 [file Data_Sheet_1.PDF]

Supplementary Material

Metagenomic Analysis of the Microbial Communities and Resistomes of Veal Calf Feces

Serajus Salaheen, Seon Woo Kim, Ernest Hovingh, Jo Ann S. Van Kessel, Bradd J. Haley

*** Correspondence:** Bradd J Haley: bradd.haley@usda.gov

Supplementary Figure 1. Non-metric multidimensional scaling (NMDS) plots based on Bray-Curtis distance matrix encompassing 12 datasets at each of the two stages of veal production. Different line colors (green, red, blue and black) represent individual farms. Panel A: NMDS plot of fecal microbial communities of veal calves at early stage of production at genus level; Panel B: NMDS plot of fecal microbial communities of veal calves at late stage of production at genus level; Panel C: NMDS plot of fecal resistomes of veal calves at early stage of production; Panel D: NMDS plot of fecal resistomes of veal calves at late stage of production. Note: In this study, veal calf management protocols were not made available. Age of the calves from different farms during each sampling may have been different.

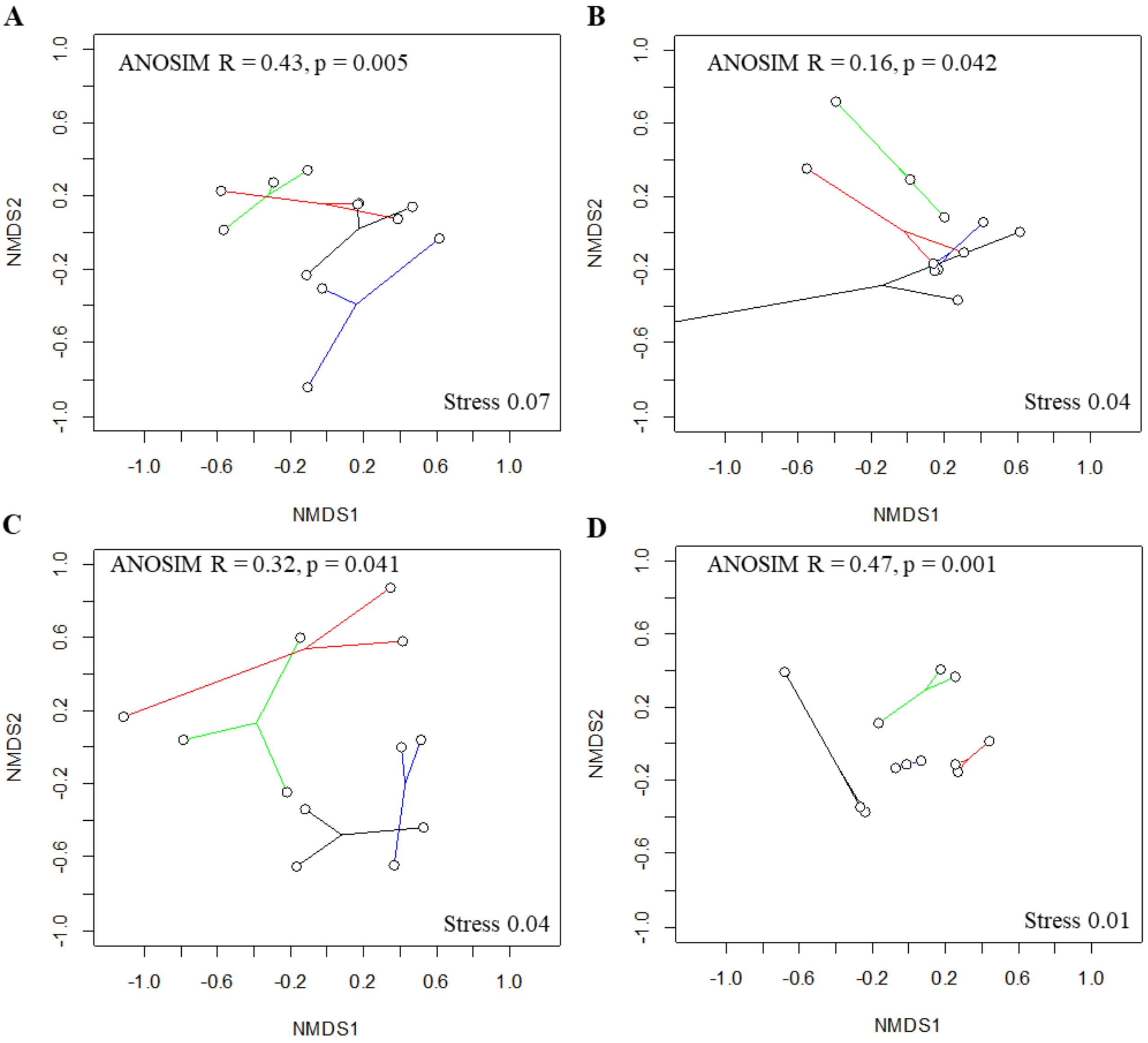
A
ANOSIM R = 0.43, p = 0.005
Stress 0.07
B
ANOSIM R = 0.16, p = 0.042
Stress 0.04
C
ANOSIM R = 0.32, p = 0.041
Stress 0.04
D
ANOSIM R = 0.47, p = 0.001
Stress 0.01
NMDS1
NMDS2
-1.0 -0.6 -0.2 0.2 0.6 1.0
