# Supplementary material for: Metagenomic Analysis of the Microbial Communities and Resistomes of Veal Calf Feces
Source: Front Microbiol. 2021 Feb 9;11:609950. doi: 10.3389/fmicb.2020.609950 (PMC7899987; doi:10.3389/fmicb.2020.609950)
Supplement: Supplementary file 2 [file Data_Sheet_2.PDF]

Supplementary Material

Metagenomic Analysis of the Microbial Communities and Resistomes of Veal Calf Feces

Serajus Salaheen, Seon Woo Kim, Ernest Hovingh, Jo Ann S. Van Kessel, Bradd J. Haley

*** Correspondence:** Bradd J Haley: bradd.haley@usda.gov

Supplementary Figure 2. Differentially abundant genera in feces collected from veal calves soon after they were brought into the farms (Sampling 1/S1, n=12) and fecal samples taken from the same cohort of calves at slaughter age (Sampling 2/S2, n=12) using linear discriminant analysis coupled with effect size measurements

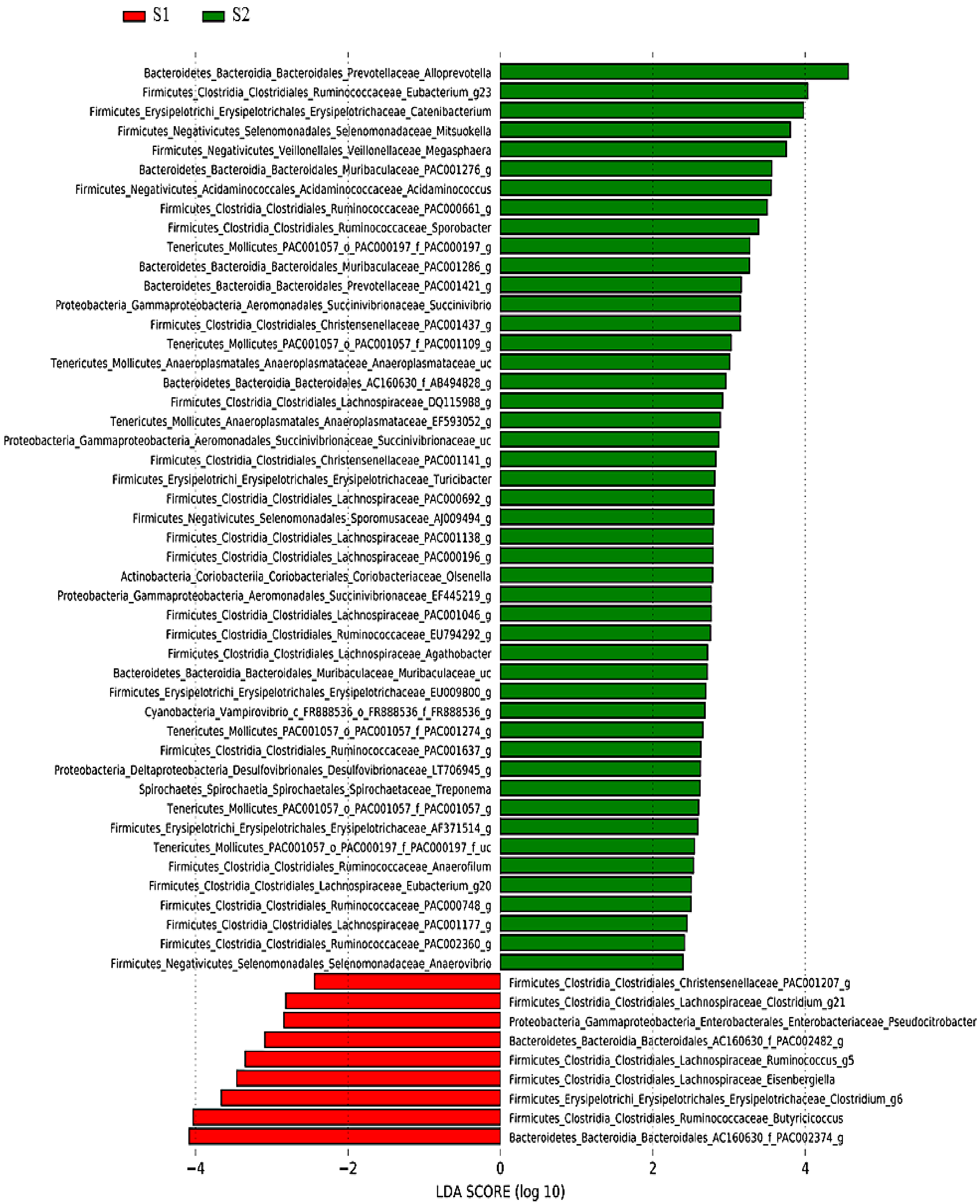
S1
S2
Bacteroidetes_Bacteroidia_Bacteroidales_Prevotellaceae_Alloprevotella
Firmicutes_Clostridia_Clostridiales_Ruminococcaceae_Eubacterium_g23
Firmicutes_Erysipelotrichi_Erysipelotrichales_Erysipelotrichaceae_Catenibacterium
Firmicutes_Negativicutes_Selenomonadales_Selenomonadaceae_Mitsuokella
Firmicutes_Negativicutes_Veillonellales_Veillonellaceae_Megasphaera
Bacteroidetes_Bacteroidia_Bacteroidales_Muribaculaceae_PAC001276_g
Firmicutes_Negativicutes_Acidaminococcales_Acidaminococcaceae_Acidaminococcus
Firmicutes_Clostridia_Clostridiales_Ruminococcaceae_PAC000661_g
Firmicutes_Clostridia_Clostridiales_Ruminococcaceae_Sporobacter
Tenericutes_Mollicutes_PAC001057_o_PAC000197_f_PAC000197_g
Bacteroidetes_Bacteroidia_Bacteroidales_Muribaculaceae_PAC001286_g
Bacteroidetes_Bacteroidia_Bacteroidales_Prevotellaceae_PAC001421_g
Proteobacteria_Gammaproteobacteria_Aeromonadales_Succinivibrionaceae_Succinivibrio
Firmicutes_Clostridia_Clostridiales_Christensenellaceae_PAC001437_g
Tenericutes_Mollicutes_PAC001057_o_PAC001057_f_PAC001109_g
Tenericutes_Mollicutes_Anaeroplasmatales_Anaeroplasmataceae_Anaeroplasmataceae_uc
Bacteroidetes_Bacteroidia_Bacteroidales_AC160630_f_AB494828_g
Firmicutes_Clostridia_Clostridiales_Lachnospiraceae_DQ115988_g
Tenericutes_Mollicutes_Anaeroplasmatales_Anaeroplasmataceae_EF593052_g
Proteobacteria_Gammaproteobacteria_Aeromonadales_Succinivibrionaceae_Succinivibrionaceae_uc
Firmicutes_Clostridia_Clostridiales_Christensenellaceae_PAC001141_g
Firmicutes_Erysipelotrichi_Erysipelotrichales_Erysipelotrichaceae_Turicibacter
Firmicutes_Clostridia_Clostridiales_Lachnospiraceae_PAC000692_g
Firmicutes_Negativicutes_Selenomonadales_Sporomusaceae_AJ009494_g
Firmicutes_Clostridia_Clostridiales_Lachnospiraceae_PAC001138_g
Firmicutes_Clostridia_Clostridiales_Lachnospiraceae_PAC000196_g
Actinobacteria_Coriobacteriia_Coriobacteriales_Coriobacteriaceae_Olsenella
Proteobacteria_Gammaproteobacteria_Aeromonadales_Succinivibrionaceae_EF445219_g
Firmicutes_Clostridia_Clostridiales_Lachnospiraceae_PAC001046_g
Firmicutes_Clostridia_Clostridiales_Ruminococcaceae_EU794292_g
Firmicutes_Clostridia_Clostridiales_Lachnospiraceae_Agathobacter
Bacteroidetes_Bacteroidia_Bacteroidales_Muribaculaceae_Muribaculaceae_uc
Firmicutes_Erysipelotrichi_Erysipelotrichales_Erysipelotrichaceae_EU009800_g
Cyanobacteria_Vampirovibrio_c_FR888536_o_FR888536_f_FR888536_g
Tenericutes_Mollicutes_PAC001057_o_PAC001057_f_PAC001274_g
Firmicutes_Clostridia_Clostridiales_Ruminococcaceae_PAC001637_g
Proteobacteria_Deltaproteobacteria_Desulfovibrionales_Desulfovibrionaceae_LT706945_g
Spirochaetes_Spirochaetia_Spirochaetales_Spirochaetaceae_Treponema
Tenericutes_Mollicutes_PAC001057_o_PAC001057_f_PAC001057_g
Firmicutes_Erysipelotrichi_Erysipelotrichales_Erysipelotrichaceae_AF371514_g
Tenericutes_Mollicutes_PAC001057_o_PAC000197_f_PAC000197_f_uc
Firmicutes_Clostridia_Clostridiales_Ruminococcaceae_Anaerofilum
Firmicutes_Clostridia_Clostridiales_Lachnospiraceae_Eubacterium_g20
Firmicutes_Clostridia_Clostridiales_Ruminococcaceae_PAC000748_g
Firmicutes_Clostridia_Clostridiales_Lachnospiraceae_PAC001177_g
Firmicutes_Clostridia_Clostridiales_Ruminococcaceae_PAC002360_g
Firmicutes_Negativicutes_Selenomonadales_Selenomonadaceae_Anaerovibrio
Firmicutes_Clostridia_Clostridiales_Christensenellaceae_PAC001207_g
Firmicutes_Clostridia_Clostridiales_Lachnospiraceae_Clostridium_g21
Proteobacteria_Gammaproteobacteria_Enterobacterales_Enterobacteriaceae_Pseudocitrobacter
Bacteroidetes_Bacteroidia_Bacteroidales_AC160630_f_PAC002482_g
Firmicutes_Clostridia_Clostridiales_Lachnospiraceae_Ruminococcus_g5
Firmicutes_Clostridia_Clostridiales_Lachnospiraceae_Eisenbergiella
Firmicutes_Erysipelotrichi_Erysipelotrichales_Erysipelotrichaceae_Clostridium_g6
Firmicutes_Clostridia_Clostridiales_Ruminococcaceae_Butyricicoccus
Bacteroidetes_Bacteroidia_Bacteroidales_AC160630_f_PAC002374_g
−4
−2
0
2
4
LDA SCORE (log 10)
